# Increasing Demand for Chest CT Due to COVID-19 in Brazil

**DOI:** 10.1590/0037-8682-0608-2020

**Published:** 2020-10-21

**Authors:** Creuza Rachel Vicente, Mariana Coelho Silva Sant'Ana, Aline Pinto Reis, Carla Fanchiotti Costa, Crispim Cerutti, Caio Martins Lanes, Geyzzara Oliveira Ferreira Diniz, Iuri Drumond Louro

**Affiliations:** 1 Universidade Federal do Espírito Santo, Departamento de Medicina Social, Vitória, ES, Brasil.; 2 Telelaudo, Vitória, ES, Brasil.; 3 The Masters School, New York, NY, United States of America.; 4 Universidade Federal do Espírito Santo, Departamento de Ciências Biológicas, Vitória, ES, Brasil.

Dear Editor:

Diagnosis of coronavirus disease 2019 (COVID-19) has been challenging since the identification of this new human infection, especially after it spread to most countries and territories of the world in the following months, increasing the worldwide demand for reverse-transcriptase polymerase chain reaction (RT-PCR) tests, the laboratory test which confirms the infection. Therefore, in settings where this test is unavailable, or results are delayed or negative in the presence of symptoms suggestive of COVID-19, chest imaging has been used in the diagnosis, clinical evaluation, and management of suspected cases^1^. Chest computed tomography (CT) contributes to a lower diagnostic loss^2^, presenting a sensitivity of 92% (95% confidence interval, 86-96%)^3^, and is recommended even in cases with a positive RT-PCR test if the patient presents mild, moderate, or severe symptoms of the disease^1^. Consensus shows some typical characteristics of images in COVID-19 cases, such as bilateral (multilobar) and peripheral opacities with an aspect of ground glass, with or without consolidations or interlobular and intralobular septal thickening (mosaic paving), multifocal and round ground-glass opacities with or without mosaic paving, and reverse halo sign^4^ ^,^ ^5^.

Since the beginning of the epidemic in Brazil on February 26, 2020, the demand for chest CT has increased, possibly due to the long wait times for the release of RT-PCR test results in some regions^6^ thereby affecting the application of the results in the clinical management of the patients. In 94 hospitals and diagnostic centers from 14 Brazilian states, serviced by Telelaudo, an increase of 192% in the demand for chest CT was observed from March 1 to June 30, 2020 (n = 14,111) when compared to the same period in 2019 (n = 4,837); increases of more than 1,000% were seen in the states of Pará in May and June, and Ceará in June. States from the North (Amazonas and Pará) and the Northeast (Ceará, Pernambuco, and Sergipe) regions had the highest increases in chest CT. These regions placed health systems in a critical situation^7^, with collapse occurring in some states, such as Amazonas^8^ and Ceará^9^ (Table 1). Especially in territories with a difficult epidemic situation, chest CT can be an effective, convenient, and fast method to recognize COVID-19 cases, thus supporting the containment of the epidemic^3^. Nevertheless, further preventive measures must be adopted in addition to the diagnosis and isolation of cases. This survey shows the impact of COVID-19 on the health services in Brazil, particularly high demand for diagnostic imaging tests to support the clinical response to the disease, in a severe epidemiological scenario which had the second-highest number of incident cases and deaths in the world in August 2020^10^.

**TABLE 1:** Number of requests for chest computed tomography by hospitals of Brazilian states from January to June, 2019 and 2020

State	March			April			May			June		
	2019	2020	Difference	2019	2020	Difference	2019	2020	Difference	2019	2020	Difference
**North region**												
Amazonas	92	203	+121%	118	1169	+891%	123	993	+707%	150	245	+63%
Pará	31	48	+55%	39	89	+128%	46	788	+1613%	60	1172	+1853%
Northeast region												
Bahia	92	93	+1%	126	143	+13%	120	335	+179%	121	225	+86%
Ceará	49	76	+55%	67	147	+119%	62	315	+408%	31	435	+1303%
Paraíba	75	62	-17%	64	88	+38%	80	169	+111%	66	71	+8%
Pernambuco	35	49	+40%	39	170	+336%	39	368	+844%	50	184	+268%
Sergipe	14	40	+186%	31	59	+90%	17	171	+906%	15	116	+673%
Midwest region												
Distrito Federal	45	153	+240%	54	120	+122%	65	179	+175%	67	133	+99%
Goiás	52	103	+98%	92	66	-28%	93	108	+16%	76	47	-38%
Mato Grosso	104	161	+55%	117	60	-49%	136	174	+28%	119	603	+407%
Southeast region												
Minas Gerais	119	192	+61%	116	315	+172%	128	328	+156%	131	258	+97%
Rio de Janeiro	205	292	+42%	219	668	+205%	322	715	+122%	228	223	-2%
São Paulo	107	200	+87%	88	309	+251%	123	338	+175%	134	195	+46%
South region												
Paraná	10	17	+70%	9	23	+156%	14	36	+157%	12	72	+500%
